# Immunohistochemical expression of the hepatocyte growth factor in chromophobe renal cell carcinoma

**DOI:** 10.1186/s12894-023-01263-0

**Published:** 2023-05-11

**Authors:** Maximilian Erlmeier, Marie Mikuteit, Stefanie Zschäbitz, Michael Autenrieth, Wilko Weichert, Arndt Hartmann, Sandra Steffens, Franziska Erlmeier

**Affiliations:** 1 Department of Urology, München Klinik Bogenhausen, Munich, Germany; 2 Department for Rheumatology and Immunology, Hannover Medical School, Carl-Neuberg-Straße 1, 30625 Hannover, Germany; 3 Dept. of Medical Oncology, National Center of Tumor Diseases, University Hospital Heidelberg, Heidelberg, Germany; 4 Department of Urology, Technical University of Munich, Klinikum Rechts der Isar, München, Germany; 5 Institute for Pathology and Pathological Anatomy, Technical University Munich, Munich, Germany; 6 Institute of Pathology, University Hospital of Erlangen, Erlangen, Germany; 7 Department of Urology, University Hospital Münster, Münster, Germany

**Keywords:** Renal Cell Carcinoma, HGF, Chromophobe histology, Survival

## Abstract

**Background:**

The prognostic value of Hepatocyte growth factor (HGF) in non-clear cell renal cell carcinoma (RCC) is still unclear. The aim of this study is to evaluate the prognostic impact of HGF expression in a large cohort of chromophobe RCC (chRCC).

**Methods:**

Patients who underwent renal surgery due to chRCC were recruited. Clinical data was retrospectively evaluated. Tumor specimen were analyzed for HGF expression by immunohistochemistry.

**Results:**

81 chRCC patients were eligible for analysis, thereof 37 (45.7%) patients were positive for HGF. No significant associations were found for HGF expression and clinical attributes in patients with chRCC. Kaplan-Meier analysis revealed no differences in 5-year overall survival (OS) for patients with HGF^−^ compared to HGF^+^ tumors (95.0% versus 90.9%; p = 0.410).

**Conclusions:**

In chRCC HGF expression is not associated with parameters of aggressiveness or survival.

## Background

Hepatocyte growth factor (HGF), also known as scatter factor, is the natural endogenous ligand of the Mesenchymal-epithelial transition factor (MET) receptor. The HGF/MET pathway is known to be associated with the appearance of several attributes of cancer and is utilized as a relevant target across many solid tumors [1]. To create active HGF, enzymes such as serum HGF activator and cellular type II transmembrane serine proteinases proteolytically cleave pro-HGF at the Arg494-Val495 bond [2]. The result is a bioactive form of HGF, a high-affinity ligand for the MET receptor: αβ-HGF. The structure of αβ-HGF shows a disulfide-linked heterodimer of an α-chain (69 kD) with β-chain subunits (34 kD) [3, 4]. Pro-HGF also binds the MET receptor, but only with very low affinity compared to αβ-HGF [5]. Only αβ-HGF can lead to overactivation of HGF/MET axis. This overactivation is known to result in tumor progression and tumorigenesis in various cancer types. Several clinical studies have addressed the role of the HGF/MET pathway in diverse types of cancer, such as gastric [6], colorectal [7], breast [8], hepatocellular [9], pancreatic [10], lung [11] and renal cancer [12]. It is a promising approach to use HGF as a prognostic marker to identify patients who gain the most benefit from HGF/MET targeted therapies. The predictive value of MET biomarkers is indisputable, as shown in several studies. High predictive values for response to foretinib and savolitinib in papillary renal cell carcinoma could be verified [13].

A broad spectrum of histopathological entities in renal cell carcinomas (RCC) is described in the 2022 World Health Organization (WHO) classification, which shows the Chromophobe renal cell carcinoma (chRCC) being the third most common (5–7%) of RCCs [14]. The chRCC shows a favourable prognosis, with high 5-year recurrence-free survival (RFS), and 10-year cancer-specific survival (CSS) [15]. Despite the fact that there is a low risk of tumor progression in patients with chRCC, some patients show development of metastatic disease with poor prognosis. There is no grading system established for this tumor subtype and due to its innate nuclear atypia, it cannot be graded by the Fuhrman grading system [14]. So far, no prognostic biomarkers exist in chRCC, leaving tumor stage, necrosis and sarcomatoid change as the most important prognostic factor for the chRCC.

Therefore, the aim of this study was to evaluate the prognostic impact of HGF in chRCC. To the best of our knowledge, this is the first study which analyzed this aspect in the third most common RCC subtype.

## Methods

### Patients and tumor characteristics

For this study 81 patients were included, who were diagnosed with chRCC after surgery by using the electronic pathology register between 1996 and 2014. Relevant clinical attributes relating to each tissue sample were collected with regard to tumor stage and histological subtype according to the Union internationale contre le cancer (UICC) 2010 TNM tumor staging system. Suitable specimens were selected by a pathologist (FE) and tissue micro arrays (TMA) were prepared from the primary tumor as previously described [16]. The histological subtype was confirmed by a second uropathologist (AH) after performing immunohistochemistry (IHC; *CD177 and CK7)*. Follow up data was inquired from electronic patient charts. To confirm overall survival (OS), data was verified by the Munich Cancer Registry of the Munich Tumor Center. The Ethics Committee of the Technical University of Munich (384/13) authorized this study with due regard to the German Human Research Act and the Declaration of Helsinki.

### Procedures

Expression of HGF was determined by IHC. 2 μm TMA slides were stained for HGF (R&D Systems, AF-294-NA, dilution 1:40) with a fully automated Dako Autostainer (Dako, Agilent pathology systems). For antigen retrieval a pH of 7.2 was accomplished. For the visualization of bound primary antibody EnVision Detection Kit (Dako, EnVision + System-HRP) was used. Therefore, sections were rinsed in tap water, counterstained with Mayer’s Hematoxylin solution and finally mounted. As positive control paraffin-embedded human colon tissue was used. All stained tissue samples were assessed in an anonymized way by a pathologist (FE). The evaluation was performed under a Leitz ARISTOPLAN light microscope (Leica Microsystems, Germany) with a x10 eyepiece, a 22-mm field of view and x40 objective lens (Plan FLUOTAR x40/0.70).

The staining reaction was classified on all available tumor surface according to a semi-quantitative IHC reference scale previously described [17]. HGF was localized primarily on the membrane and partly in the cytoplasm of tumor cells. Tissue with sarcomatoid change, growth pattern, and necrosis were not punched.

**Figure 1:**
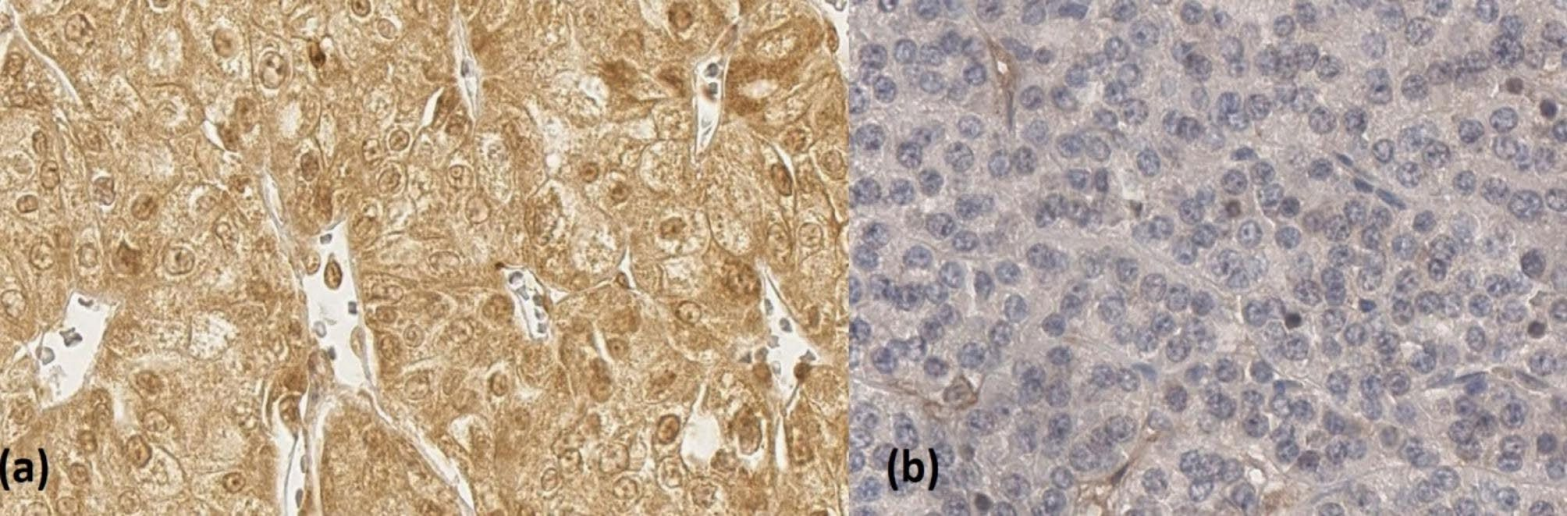
Immunohistochemical staining of HGF in chromophobe renal cell carcinoma specimen. (**a**) positive (40x magnification) (**b**) negative (40x magnification)

The staining intensity was scored from 0 to 3 (0 = no staining, 1 = weak staining (pale yellow), 2 = moderate staining (yellowish brown), 3 = strong staining (brown)) according to the H-score as already described (Fig. 1) [18–20]. The area of staining was evaluated in percent (0-100%), a staining intensity score was defined by multiplying the score with the stained area (Table 1) [17, 21]. To dichotomize our patient collective, we defined the median of observed distribution as the cut off, since there is no normative data in the literature regarding the staining intensity of cell membrane or cell cytoplasm. Because of the limited number of cases we used the median as a binary cutoff. Since the median was 0, an HGF staining score of 0 was defined as HGF^−^, and a staining higher than 0 was defined as HGF^+^.

**Table 1:** Pathohistological characteristics of the chRCC samples (n = 81)

	Min-Max	Mean (SD)	Median (IQR)
**HGF intensity**	0–3	0.77 (1.0)	0.0 (0.0–1.0)
**HGF area**	0-100	27.78 (37.28)	0.0 (0.0–70.0)
**HGF score**	0-300	54.20 (92.69)	0.0 (0.0–70.0)

SD = standard deviation, IQR = inter quartile range

### Statistical analysis

The primary endpoint of the study was OS. In the absence of death, the endpoint was censored at the last date of follow-up. Follow-up was defined by the time span from the date of the surgery to the date of death or from surgery to the last known date of follow-up.

To analyze patient/tumor characteristics in relation to corresponding subgroups with or without HGF expression, several tests were used in correlation to the nature of the requested variable: Fisher’s exact tests, chi-square, independent t-test, and Mann-Whitney U-Test. Kaplan-Meier survival times were estimated, with subgroups being compared using the log-rank test. SPSS 27.0 (USA) was used for statistical assessment. Two-sided p-values below 0.05 were considered statistically significant.

## Results

### Patients´ characteristics and HGF expression

The median age of the cohort was 59.8 (range: 31–79) years. Of the patients 60 (74.1%), 14 (17.3%) and 7 (8.6%) presented with pT1, pT2 and pT3 tumors, respectively. 86.4% of the patients had AJCC Stage I/II. Furthermore, 6 (7.4%) of all patients presented with lymph node metastasis or synchronous distant metastasis. HGF expression was found in 37 (45.7%) of the chRCC TMA specimens, respectively (Fig. 1). No associations between HGF^+^ expression and patient or tumor characteristics (including lymph node metastasis, distant metastasis, Cancer Stage (AJCC) and T-stage) were identified (Table 2).

**Table 2:** chRCC patient´s and tumor characteristics in dependence of HGF expression

Variable	All chRCC n = 81 (100%)	HGF^-^ n = 44 (54.3%)	HGF^+^ n = 37 (45.6%)	p-value
Age, median (IQR) years	59.8 (52.9–69.1)	62.7 (51.2–71.1)	59.4 (53.2–67.6)	0.649^a^
Sex				0.472^b^
female	23 (28.4%)	11 (25.0%)	12 (32.4%)	
male	58 (71.6%)	33 (75.0%)	25 (67.6%)	
Stage (TNM 2010)				0.639^c^
pT1	60 (74.1)	34 (77.3%)	26 (70.3%)	
pT2	14 (17.3)	6 (13.6%)	8 (21.6%)	
pT3	7 (8.6)	4 (9.1%)	3 (8.1%)	
Cancer Stage (AJCC)				0.612^c^
Stage I	56 (69.1)	33 (75.0%)	23 (62.2%)	
Stage II	14 (17.3)	6 (13.6%)	8 (21.6%)	
Stage III	8 (9.9)	4 (9.1%)	4 (10.8%)	
Stage IV	3 (3.7)	1 (2.3%)	2 (5.4%)	
LN metastasis ^#^				0.590^b^
N-	78 (96.3)	43 (97.7%)	35 (94.6%)	
N+	3 (3.7)	1 (2.3%)	2 (5.4%)	
Metastasis^#^				0.590^b^
M-	78 (96.3)	43 (97.7%)	35 (94.6%)	
M+	3 (3.7)	1 (2.3%)	2 (5.4%)	
Disease status				0.537^b^
Localized *	70 (86.4)	39 (88.6%)	31 (83.8%)	
Advanced ^$^	11 (13.6)	5 (11.4%)	6 (16.2%)	

^#^ at time of renal surgery; * localized disease = pT1/2 N0/M0; ^$^ advanced disease = pT3/4 and/or N + and/or M+. Legend: IQR: Interquartile range, NE: not evaluable; N- = lymph node status unknown or tumour cells absent from regional lymph nodes, N + = regional lymph node metastasis present. ^a^ Mann-Whitney-U test, ^b^ Fisher exact test, ^c^ chi square test

### HGF expression and clinical course

Median follow-up was 40.5 (IQR: 10.8-109.3) months. At the time of last follow-up, 46 (56.8%) patients were alive, 9 (11.1%) patients died and 26 (32.1%) patients were lost to follow up. In the subgroups of HGF^−^ vs. HGF^+^ 25 (56.8%) vs. 21 (56.8%) patients were alive, 3 (6.8%) vs. 6 (16.2%) patients had died and 16 (36.4%) vs. 10 (27.0%) patients were lost to follow up (p = 0.342, chi square).

**Figure 2:**
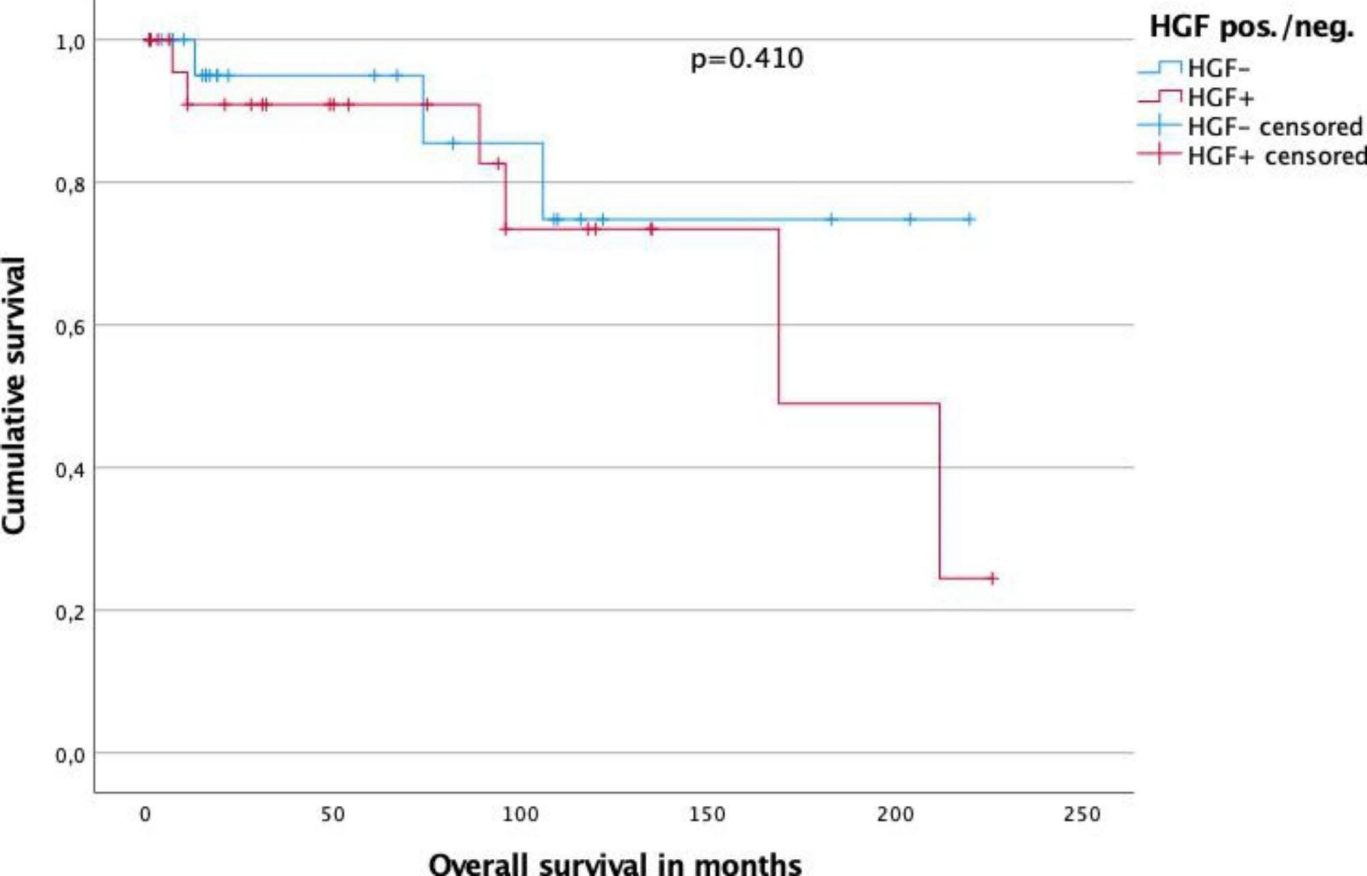
5-year overall survival for patients with chromophobe renal cell carcinoma in dependence of HGF expression. Kaplan-Meier analysis disclosed a 5 year- OS for HGF^-^ compared to HGF^+^ tumors of 95.0% compared to 90.9% (p = 0.410, log rank) (Fig. 2)

Kaplan-Meier analysis disclosed a 5 year- OS for HGF^−^ compared to HGF^+^ tumors of 95.0% compared to 90.9% (p = 0.410, log rank) (Fig. 2).

## Discussion

To the best of our knowledge, this is the first study which analyzed the prognostic role of HGF expression in chRCC. In view of the fact that the HGF/MET pathway is relevant in tumor progression and tumorigenesis in diverse types of cancer [6–12, 22], it seems obvious that therapeutic approaches are expected to target either the ligand (HGF) or the receptor (MET). There are already available therapeutic options targeting MET (c-MET inhibitors, e.g. Cabozantinib, Crizotinib and Capmatinib) [23]. The study by Silva Paiva et al. relates to c-Met expression in renal cell carcinoma with bone metastases and shows a detailed overview of therapeutic options, targeting the c-Met pathway [22]. Despite the strong pre-clinical evidence that simultaneous targeting of both the ligand and the receptor, offers the most effective approach for reducing cancer, clinical investigations regarding this setting are rare. Recently, promising pre-clinical results have led to clinical trials in patients with refractory solid tumors using YYB101, a HGF neutralising antibody [24]. YYB102 leads to almost total inhibition of the HGF/MET pathway by binding to the alpha chain of HGF resulting in thoroughly blocking HGF from binding c-MET [24]. However, there are no clinical trials using YYB101 in RCC. Clinical trials are currently limited to Phase 1b/2a clinical trial in metastatic colorectal cancer patients in Korea [24]. The focus of our study lays on HGF as a promising prognostic marker, knowing that the HGF/MET pathway is one of the most important pro-oncogenic, pro-angiogenetic, and pro-metastatic signals in various cancer types [25]. Despite promising pre-clinical evidence regarding the HGF/MET pathway in numerous solid types of cancer, results in our study show a surprisingly low clinical impact of HGF expression in chRCC.

Several evidences have shown that HGF/Met signaling is substantial in cancer cells for the maintenance of self-renewal mechanisms and even development of chemoresistance [26]. Currently, research results indicated that HGF/Met signaling could support mechanisms for immune escape of cancer cells, so new approaches are being developed, to combine MET and programmed cell death receptor-1 (PD-1)/programmed cell death receptor-ligand 1 (PD-L1) inhibition in drug design and targeted therapy [25, 26].

There are of course several limitations evaluating these results: the study design uses a retrospective analysis. Due to the rare incidence of this tumor subtype the number of cases is limited, however, 81 chRCC cases can be considered as a large cohort. Additional limitations are the use of TMAs and the methodology of immunohistochemistry.

## Conclusions

In conclusion our study aimed to evaluate HGF as a prognostic marker in chRCC, using a large group of 81 (26 lost to follow-up) patients diagnosed with chRCC. However, HGF did not qualify as a prognostic marker in chRCC for survival.

## Data Availability

The datasets generated and/or analyzed during the current study are not publicly available due to medical confidentiality regarding patients’ data but are available from the corresponding author on reasonable request.

## Abbreviations

AJCC: American Joint Committee on Cancer
chRCC: Chromophobe renal cell carcinoma
CSS: Cancer specific survival
HGF: hepatocyte growth factor
IHC: Immunohistochemistry
MET: Mesenchymal-epithelial transition factor
OS: Overall survival
RCC: Renal cell carcinoma
TMA: Tissue micro arrays
UICC: Union internationale contre le cancer
WHO: World Health Organization
